# Obituary

**Published:** 1893-05

**Authors:** 


### Obituary.

Dr. Edward L. Gager, of Buffalo, died very suddenly of edema of the glottis at the Warsaw Sanitarium, Tuesday, March 28, 1893. Dr. Gager was a graduate of the University of Buffalo, and was thoroughly fitted for his work, but for some reason he abandoned his practice and entered mercantile life a few years ago. His illness was indirectly due to exposure while doing military duty during the railroad strike of last summer.

Dr. William Pierce Seymour, of Troy, N. Y., died at his residence in that city on Friday, April 7, 1893. He was born in Troy, October 17, 1825, and had been a practitioner of medicine in the city of his birth since 1848. He was formerly professor of Materia Medica, Obstetrics, and Diseases of Women in the Berkshire Medical College at Pittsfield, Mass., and of Obstetrics and Gynecology in the Albany Medical College, and the University of Vermont. He was a member of the Rensselaer County Medical Society, the Medical Society of the State of New York, and the American Medical Association.

Dr. Seymour was a man of culture and education, eminent in his profession, and a progressive man in every sense of the word. His loss is a serious one not only to the profession, but to his family and a wide circle of intimate friends and acquaintances. Dr. William Wotkyns Seymour, his eldest son, is also a physician of eminence who will maintain the distinction of the family name. Another son, Mr. Alfred Wotkyns Seymour, resides in Chicago.

Dr. Seymour's funeral was held on Monday, April 10th, and in accordance with the expressed wish of the deceased, the remains were cremated.

